# Acute chest syndrome and COVID‐19 in hydroxyurea naïve sickle cell disease patient in a low resource setting

**DOI:** 10.1002/jha2.397

**Published:** 2022-02-07

**Authors:** William Frank Mawalla, Ahlam Nasser, James Salumu Jingu, Happiness Joseph, Lilian Gasper Mmbaga, Eunice Shija, Helena Kakumbula, Neema Budodi Lubuva, Collins Meda, Clara Chamba

**Affiliations:** ^1^ Department of Haematology and Blood Transfusion Muhimbili University of Health and Allied Sciences Dar es Salaam Tanzania; ^2^ Department of Internal Medicine Muhimbili National Hospital Dar es Salaam Tanzania

**Keywords:** acute chest syndrome, case report, COVID 19, hydroxyurea, SARS‐Cov‐2, sickle cell disease

## Abstract

Acute chest syndrome (ACS) is a severe complication of sickle cell disease (SCD) and one of the leading causes of mortality in SCD patients. The management of ACS is challenging and requires prompt intervention to halt clinical deterioration. With the outbreak of the Coronavirus Disease 2019 (COVID‐19) pandemic, which also primarily results in acute respiratory illness, the clinical picture and treatment outcome in SCD patients with ACS remain unknown. We present a case of a 30‐year‐old male who came in with features of painful vaso‐occlusive episode and haemolysis that later evolved to acute chest syndrome. Chest X‐ray showed pneumonic changes and mild bilateral pleural effusion, and nasal Reverse Transcription‐Polymerase Chain Reaction (RT‐PCR) for COVID‐19 test came out positive. He was managed supportively with simple transfusion, antibiotics, dexamethasone and oxygen support with a good clinical outcome. Presenting with non‐specific symptoms and similar respiratory symptoms and signs, the clinical picture of COVID‐19 can prove difficult to discern from that of ACS due to other causes. This report emphasizes a need for a higher index of suspicion whenever a SCD patient presents with symptoms of acute respiratory distress.

**List of Abbreviations**
ACS: acute chest syndrome
COVID‐19: corona virus disease 2019
Hb: haemoglobin
MNH: Muhimbili National Hospital
MUHAS: Muhimbili University of Health and Allied Sciences
SCA: sickle cell anaemia
SCD: sickle cell disease
VOC: vaso‐occlusive crisis

## 1 INTRODUCTION

Acute chest syndrome (ACS) is a severe complication of sickle cell disease (SCD) and one of the leading causes of mortality in SCD patients [1, 2]. The clinical presentation is of severe respiratory failure with higher mortality in adults than in children [2, 3]. Even with advanced healthcare systems and the best clinical settings, the management of ACS is challenging and requires prompt intervention to halt clinical deterioration [4]. The picture is even more complicated in low‐income regions such as Sub‐Saharan Africa, where over 75% of the 400,000 infants born annually with the disease globally originate [5, 6, 7]. With the outbreak of the COVID‐19 pandemic, which also primarily results in acute respiratory illness, the presentation and treatment outcome of ACS in SCD patients remain unknown. COVID‐19 pandemic is, therefore, a cause of great concern in the already fragile healthcare systems in low‐income regions.

We present this case to highlight the challenges in diagnosing and managing ACS in SCD patients with Severe Acute Respiratory Syndrome Coronavirus 2 (SARS‐CoV‐2) infection in a setting of limited healthcare resources.

## 2 CASE DESCRIPTION

A 30‐year‐old male, a resident of Dar es Salaam, Tanzania, with a known history of sickle cell anaemia (HbSS), was referred to our hospital with fever and generalized bone pains. The fevers were of low grade, associated with excessive night sweats, and subsided with paracetamol. The onset of the bone pains was gradual, beginning as mild back pain before involving the extremities and progressing into a sharp, persistent pain. No report of any respiratory symptoms was noted before the current admission. However, the patient reported a history of upper right abdominal pain, which had been previously diagnosed as gall stones at a primary healthcare facility and was managed conservatively with oral medications with some improvement. The abdominal pain was not associated with nausea, vomiting, or diarrhoea, and bowel motions and micturition habits were normal.

The patient's steady‐state haemoglobin levels were unknown as he was not attending a regular sickle cell clinic. He was taking daily folic acid tablets and had never used hydroxyurea. Nevertheless, he reports no history of hospital admission in the past 2 years, despite having reported few episodes of vaso‐occlusive pain events that were managed in outpatient care settings. Further, he reports no history of previous blood transfusion and received all vaccines according to the local immunization scheme, including pneumococcal.

At presentation, physical examination revealed the patient was febrile (39.1°C) with moderate pallor, sclera jaundice, and no palpable lymph nodes. He had a respiratory rate of 22 breaths per minute, and chest examination revealed: normal chest shape and movement, resonant note on percussion, and vesicular breath sounds over regions of the anterior and posterior chest. Finger pulse oximetry oxygen saturation was 96% on room air. The rest of the systemic examination was essentially normal.

**FIGURE 1:**
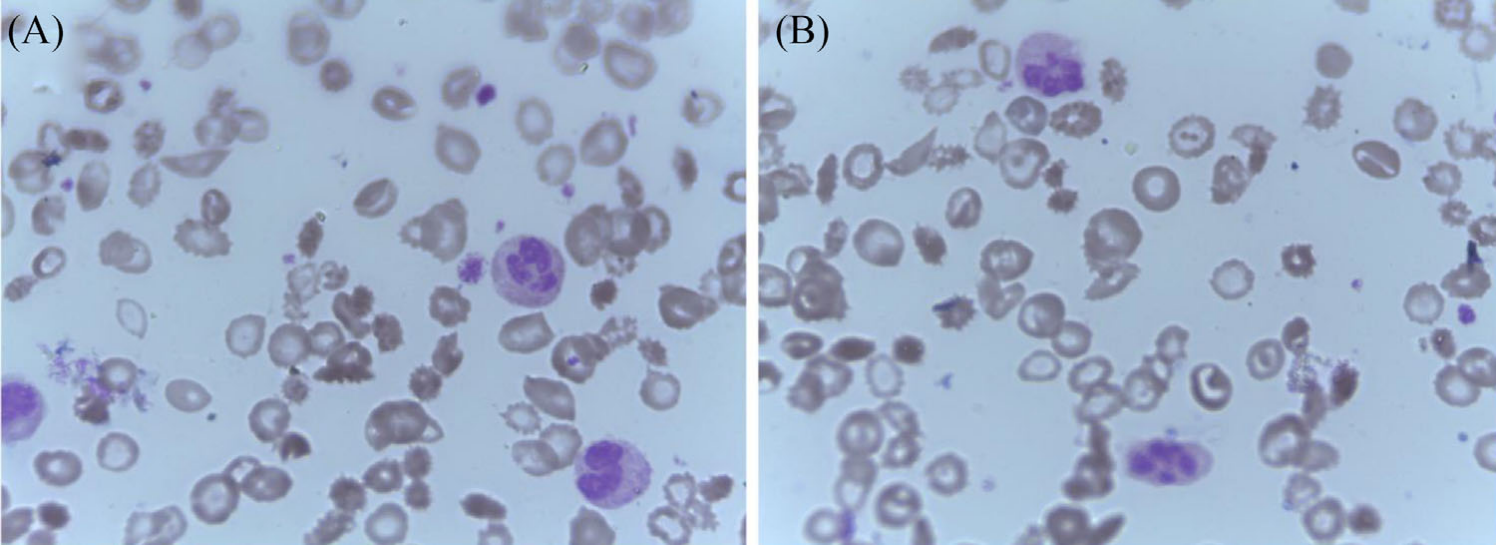
(A and B) Light microscopy of peripheral blood smear with field sections showing anisopoikolocytosis and a few sickle‐like red cells. The slide also shows neutrophils with toxic granulations

The blood count at presentation showed leukocytosis, microcytic anaemia, and normal platelet count. The reticulocyte count was significantly raised (Table 1). The peripheral blood smear showed sickled red blood cells, neutrophilia with a left shift and toxic granulation; features consistent with sickle cell anaemia and an ongoing infective process (Figure 1A,B). Total and unconjugated bilirubin, lactate dehydrogenase and liver enzymes were all raised. Urea and creatinine levels were within the normal range. The rapid malaria antibody test was negative (Table 2). The proportion of sickle or fetal haemoglobin levels could not be measured due to the unavailability of the test at our facility at the time of admission.

**TABLE 1:** Patient's complete blood count

Parameter	Reference range	Baseline	Day 14	Day 41
White blood count, total, (per L)	4–10 × 10^9^	22.68 × 10^9^	8.85 × 10^9^	8.61 × 10^9^
Neutrophil, (per L)	2–6.9 × 10^9^	17.68 × 10^9^	4.48 × 10^9^	3.59 × 10^9^
Lymphocyte, (per L)	0.6–3.4 × 10^9^	4.0 × 10^9^	3.41 × 10^9^	4.20 × 10^9^
Monocyte, (per L)	0.0–0.9 × 10^9^	5.3 × 10^9^	0.74 × 10^9^	0.58 × 10^9^
Basophil, (per L)	0.0–2 × 10^9^	0.16 × 10^9^	0.1 × 10^9^	0.16 × 10^9^
Eosinophil, (per L)	0.0–0.7 × 10^9^	0.03 × 10^9^	0.1 × 10^9^	0.06 × 10^9^
Haemoglobin	13–17	6.60	8.96	8.50
MCV (fl)	83–99	75.2	87.54	87.62
Mean Corpuscular Volume (MCH) (pg)	27–32	24	25.44	25.50
Mean Corpuscular Haemoglobin Concentration (MCHC) (g/L)	315–345	329	296	290
RDW (%)	11.6–14.8	31.79	21.37	20.35
Platelets (per L)	150–410 × 10^9^	169.3 × 10^9^	598.5 × 10^9^	572.7 × 10^9^
Reticulocyte count (absolute)	50–100 × 10^9^	156 × 10^9^	‐	377 × 10^9^

Abbreviations: MCV, mean cell volume; RDW, red cell distribution width.

**TABLE 2:** Other laboratory markers

Test	Reference range	Baseline	Day 14	Day 41
**Inflammatory markers**				
CRP	0–5 mg/dl	309.5 mg/dl	157.7 mg/dl	–
Serum ferritin	13–55 ng/ml	362.7 ng/ml	–	–
**Haemostasis markers**				
D‐dimer	0–0.5 µg/ml	13.0 µg/ml	12.0 µg/ml	7.41 µg/ml
Prothrombin time	9.4–12 s	12.5 s	11.7 s	–
Partial thromboplastin time	25.4–36.9 s	28 s	31.8 s	–
**Haemolysis markers**				
LDH	60–100 U/L	279 U/L	‐	–
Total bilirubin	3.4–20.5 μmol/L	38.8 μmol/L	19.4 μmol/L	16.8 μmol/L
Direct bilirubin	0–8.6 μmol/L	9 μmol/L	8.3 μmol/L	12.5 μmol/L
ASAT	5–34 U/L	387 U/L	33 U/L	53 U/L
ALAT	0–55 U/L	84 U/L	84 U/L	55 U/L
GGT	12–64 U/L	368 U/L	458 U/L	248 U/L
**Renal function tests**				
Creatinine	63.6–110.5 μmol/L	63.3 μmol/L	43.1 μmol/L	27.9 μmol/L
Urea nitrogen	3.2–7.4 μmol/L	4.69 μmol/L	1.24 μmol/L	1.66 μmol/L

Abbreviations: ALAT, alanine aminotransferase; ASAT, aspartate aminotransferase; CRP, C‐reactive protein; GGT, gamma‐glutamyl transferase; LDH, lactate dehydrogenase.

The patient was started on IV Paracetamol 1 g, 6 hourly for 2 days, and IV Ceftriaxone‐sulbactam 1.5 g 12 hourly for 5 days. Since the patient presented with fever and had signs of dehydration, we placed the patient on intravenous normal saline equivalent to 1.5 times the normal maintenance fluid dose over 24 hr. Daily folic acid was continued at 5 mg daily, and oral Hydroxyurea 1 g once daily was initiated to prevent future episodes of vaso‐occlusive events. He also received a top‐up transfusion with two units of packed red cells to reach a haemoglobin level of above 8 g/dl.

**FIGURE 2:**
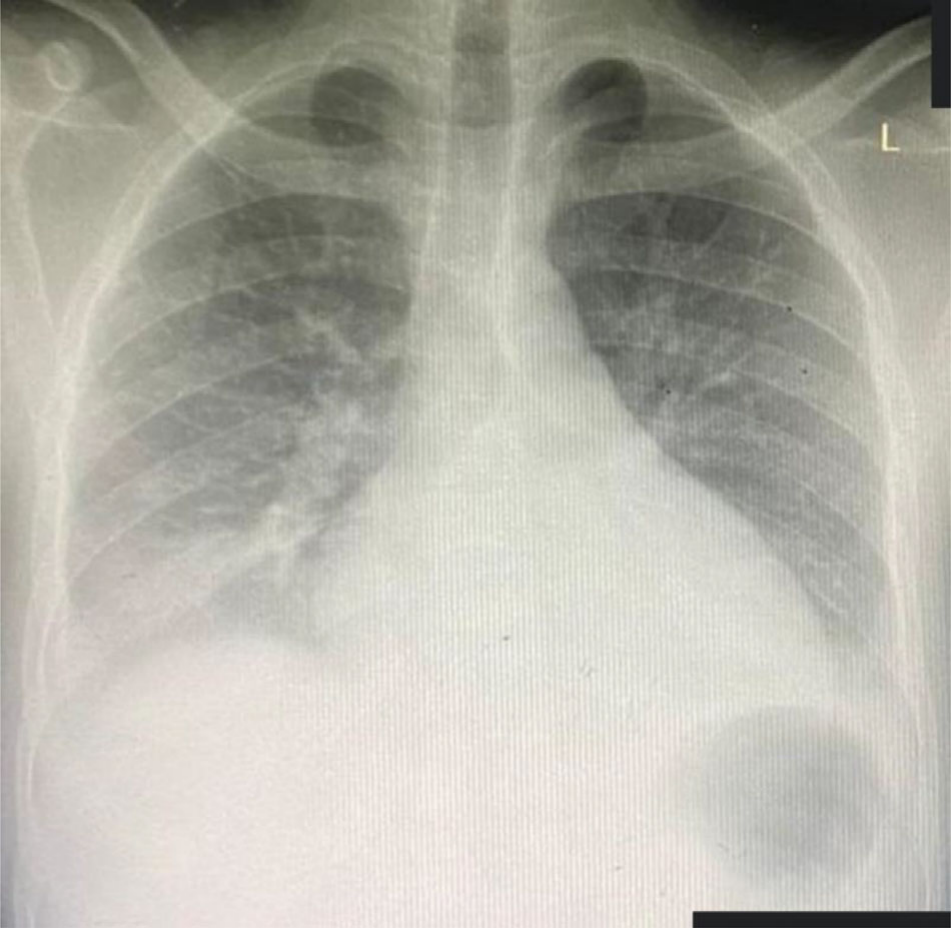
A chest X‐ray image (PA view) with features of pneumonic process with mild bilateral pleural effusion. The film shows bilateral consolidation that is more on the middle and lower zones. Vascular markings and the horizontal fissure are prominent, and the right costophrenic angle is blunted

By the third day of admission, the fever had subsided but the patient started developing difficulty in breathing and dark coloured urine. Respiratory examination revealed a dull percussion note on the right side of the chest, bilateral bronchial breath sounds and areas of coarse crackles on auscultation. Finger pulse oximetry revealed oxygen saturation of 85% at room air. Chest X‐ray showed bilateral consolidation with mild bilateral pulmonary effusion (Figure 2). A nasopharyngeal swab for the SARS‐CoV‐2 RT‐PCR test was performed, and the result was positive.

The patient was started on oxygen therapy with a non‐rebreather mask at an initial rate of 10 L/m. This improved the saturation levels to 94%–96% on oxygen. Based on the local treatment guideline for COVID‐19 patients that was available at the time patient was seen; IV dexamethasone 8 mg once daily and oral azithromycin 500 mg daily for 7 days were added to his treatment regimen. The patient was also given oral multivitamin and mineral supplements. Exchange transfusion could not be undertaken due to the inadequate capacity to conduct the procedure and to run the necessary preliminary tests that would be used to monitor the effectiveness of the exchange transfusion.

At 48 h following steroid and oxygen therapy initiation, there was an improvement in breathing and a decrease in oxygen demand to 5 L/m. The urine was also back to normal colour. On day seven, he was afebrile, the difficulty in breathing had resolved completely, and the patient was weaned off oxygen while saturating at 95%–97% on room air. At this point, the painful attacks had ceased, and symptoms of anaemia subsided. The patient was maintained on oral fluid, folic acid and hydroxyurea.

**FIGURE 3:**
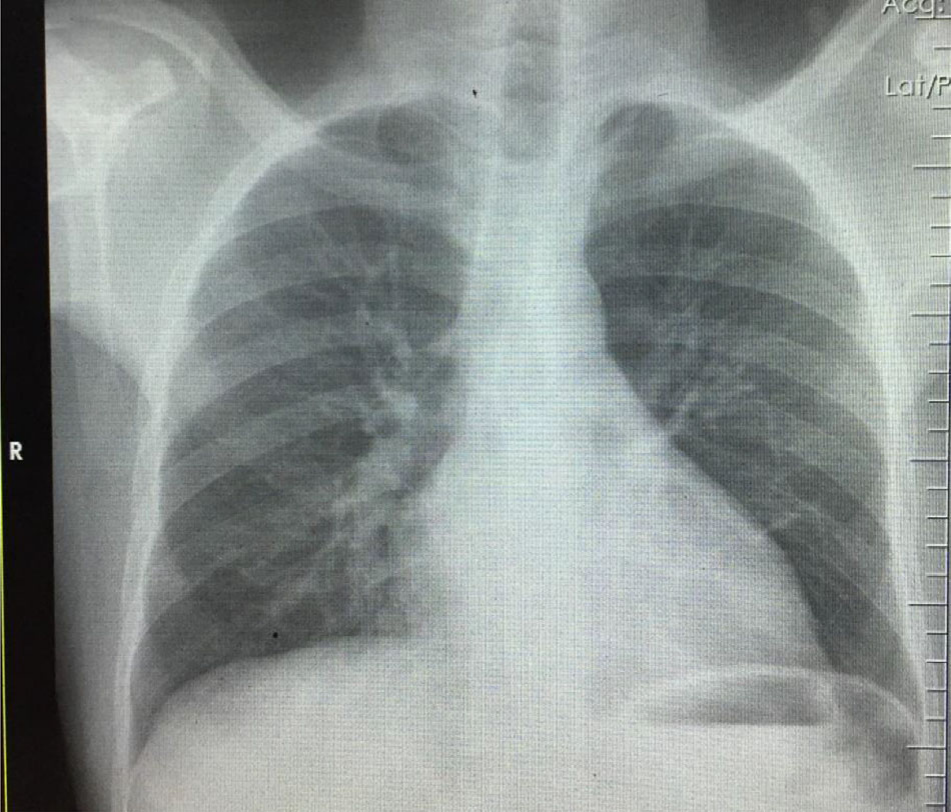
A repeat chest X‐ray image (PA view) done after 6 weeks shows significant improvement with resolved pleural effusion and decreased interstitial lung markings

On day 14, the patient had significant clinical improvement. All blood cultures had no growth. The white cell count and differential had normalized, and the haemoglobin increased to 8.96 g/dl after top‐up transfusion with two units of packed red blood cells. The C‐reactive protein (CRP) dropped by half, and the aspartate aminotransferase (ASAT) and bilirubin levels normalized. However, there was no significant drop in the D‐dimer or alanine aminotransferase levels, and gamma‐glutamyl transferase had risen slightly further (Tables 1 and 2). Nevertheless, a repeat nasopharyngeal swab for SARS‐CoV‐2 was negative, and with the continued improved clinical state, he was discharged home after 17 days of hospitalization. A repeat chest X‐ray that was done after 6 weeks showed resolved features of consolidation and pleural effusion (Figure 3). Further, the patient's clinical symptoms had completely resolved and had normal respiratory examination findings.

## 3 DISCUSSION AND CONCLUSIONS

Our patient presented with features of vaso‐occlusive painful event and haemolysis with progression to acute respiratory distress that fit the criteria for ACS [8]. Pre‐COVID‐19 studies [3, 9, 10] have shown ACS is often preceded with vaso‐occlusive painful episodes and haemolytic events, similar to our patient's presentation. However, studies describing the impact of COVID‐19 in the clinical course of sickle cell disease crises are limited. Furthermore, the mechanistic interaction and effect of the COVID‐19 and ACS are poorly known, and one can only allude to a fatal synergistic effect of the two primarily respiratory diseases [7].

Few reported cases [11, 12, 13, 14, 15, 16] of SCD patients with COVID‐19 presented similarly, with features of vaso‐occlusive crisis (VOC) painful events such as fevers, limb and back pains that evolved to haemolytic anaemia and/or ACS. Some of the described ACS features include cough, dyspnea, hypoxia (low peripheral oxygen saturation) and new pneumonic changes on chest radiography [12, 13, 14, 15, 16]. While pulmonary embolism due to thrombi can be one of the causes of acute respiratory distress, the preceding features of VOC and new radiodensities on the chest X‐ray in our patient made us rule it out, even in the presence of mild elevation of D‐dimers. The laboratory parameters in reported cases of SCD patients with COVID‐19 show a wide variation [7]. Nonetheless, most cases have elevated markers of acute inflammation and haemolysis. Acute phase reactants such as CRP, ferritin, D‐dimers are non‐specific and can be elevated in both SCD crises and COVID‐19 flares [7, 17]. Attempts have been made to utilize inflammatory and haemolytic markers to predict disease severity and outcome in COVID‐19 patients [18, 19]. However, results from these studies, most of which are from non‐SCD patients, cannot be applied to the SCD population. SCD causes a relatively constant inflammatory state with deranged inflammatory and haemolytic markers that further deviate at times of vaso‐occlusive episodes [20, 21].

The baseline blood count (Table 1) in our patient showed a low mean cell volume, mean cell haemoglobin and red cell distribution width, all features suggestive of iron deficiency anaemia. We could not use serum ferritin to confirm the iron deficiency state because of the low sensitivity of these tests in patients with sickle cell anaemia (SCA) and a setting of acute infection [22]. In our case, serum ferritin, which is also an acute‐phase reactant was elevated, hence not useful in the diagnosis of iron deficiency. Transferrin saturation assay was not available for further comparison [23]. Iron deficiency though uncommon in patients with SCA has been reported in studies done in Tanzania and other regions with a high prevalence of iron‐deficiency anaemia [24, 25, 26]. The possibility of the concomitant existence of thalassemia could not be ruled out due to the lack of genetic studies.

Regarding the management of ACS, there is a lack of randomized trials to inform standard therapy. Nevertheless, transfusion (simple and/or exchange) to maintain the proportion of sickle haemoglobin below 30% and correct the severe anaemia is currently considered the mainstay [8, 27, 28]. This approach has also been employed in the few reported patients with ACS and COVID‐19, who subsequently had good clinical outcomes [12, 13, 16]. Although there has been a report of case [29] in a resource‐limited setting where manual exchange transfusion was implemented with limited blood supply with good clinical results, this could not be reproduced in our case as we had no means of testing baseline HbS levels. We, therefore, had to rely on simple top‐up transfusion.

Even though the use of systemic corticosteroids is known to trigger rebound vaso‐occlusive painful episodes in SCD patients, we nevertheless elected to administer dexamethasone in our patient after weighing the benefits of giving dexamethasone in the context of severe COVID‐19 infection versus the risk of rebound VOC [30]. The use of dexamethasone in SCD patients with severe respiratory disease due to COVID‐19 is still recommended to date and has been shown to limit severe lung injury. Rebound vaso‐occlusive events, if they occur, are in turn managed symptomatically [7, 31]. We monitored our patient closely, and he did not develop a rebound vaso‐occlusive event.

 Likewise, hydroxyurea, a crucial drug in SCD, is known to decrease the incidence of acute painful events and ACS and prolongs survival [32]. Our patient, having never used hydroxyurea, was, therefore, more vulnerable to ACS. Rather than managing the current event, hydroxyurea was initiated in our patient to reduce the risk of future vaso‐occlusive events. Whether hydroxyurea has any role in preventing any additional complications of COVID‐19 in SCD patients is yet to be determined.

The COVID‐19, being a respiratory virus, is expected to worsen an already fatal respiratory disease in the vulnerable SCD population, especially in low‐income regions where it is difficult to institute standard care management. Presenting with non‐specific symptoms, the clinical picture of COVID‐19 can prove difficult to discern from ACS due to other causes. Even in a setting of limited resources, early diagnosis and timely initiation of supportive care can result in good clinical outcomes in patients with ACS and COVID‐19. This report emphasizes a need for a higher index of suspicion whenever a SCD patient presents with symptoms of acute respiratory distress. 

## CONSENT TO PUBLICATION

Written informed consent for publication of clinical details and clinical images was obtained from the patient. A copy of the consent form is available for review by the editor.

## CONFLICT OF INTEREST

The authors have no conflict of interest to disclose.

## AUTHOR CONTRIBUTIONS

William Frank Mawalla gathered the patient's history and clinical findings, interpreted the laboratory results and wrote the report. Ahlam Nasser managed the patient and wrote the report. James Salumu Jingu collected the patient's history, conducted a clinical examination, interpreted laboratory test results, managed the patient and wrote the paper. Happiness Joseph gathered the patient's history and clinical findings, interpreted the laboratory results and wrote the report. Lilian Gasper Mmbaga gathered the patient's history and clinical findings, interpreted the laboratory results and wrote the report. Eunice Shija collected the patient's history, conducted a clinical examination, interpreted laboratory test results, managed the patient and wrote the paper. Helena Kakumbula gathered the patient's history and clinical findings, interpreted the laboratory results and wrote the report. Neema Budodi Lubuva gathered the patient's history and clinical findings, interpreted the laboratory results and wrote the report. Collins Meda reviewed the manuscript and interpreted the laboratory results. Clara Chamba managed the patient and wrote the report. All authors participated in a critical review of the final paper for submission.

## References

[1] Platt OS , Brambilla DJ , Rosse WF , Milner PF , Castro O , Steinberg MH , et al. Mortality in sickle cell disease. Life expectancy and risk factors for early death. N Engl J Med. 1994;330(23):1639–44. 7993409 10.1056/NEJM199406093302303

[2] Allareddy V , Roy A , Lee MK , Nalliah RP , Rampa S , Allareddy V , et al. Outcomes of acute chest syndrome in adult patients with sickle cell disease: predictors of mortality. PLoS One. 2014;9(4):e94387. 24740290 10.1371/journal.pone.0094387 PMC3989222

[3] Vichinsky EP , Neumayr LD , Earles AN , Williams R , Lennette ET , Dean D , et al. Causes and outcomes of the acute chest syndrome in sickle cell disease. National Acute Chest Syndrome Study Group. N Engl J Med. 2000;342(25):1855–65. 10861320 10.1056/NEJM200006223422502

[4] Jain S , Bakshi N , Krishnamurti L . Acute chest syndrome in children with sickle cell disease. Pediatr Allergy Immunol Pulmonol. 2017;30(4):191–201. https://pubmed.ncbi.nlm.nih.gov/29279787 29279787 10.1089/ped.2017.0814 PMC5733742

[5] Dexter D , Simons D , Kiyaga C , Kapata N , Ntoumi F , Kock R , et al. Mitigating the effect of the COVID‐19 pandemic on sickle cell disease services in African countries. Lancet Haematol. 2020;7(6):e430–2. 10.1016/S2352-3026(20)30122-8 32334676 PMC7180020

[6] Piel FB , Patil AP , Howes RE , Nyangiri OA , Gething PW , Dewi M , et al. Global epidemiology of sickle haemoglobin in neonates: a contemporary geostatistical model‐based map and population estimates. Lancet 2013;381(9861):142–51. 23103089 10.1016/S0140-6736(12)61229-X PMC3547249

[7] Alsayegh F , Mousa SA . Challenges in the management of sickle cell disease during SARS‐CoV‐2 pandemic. Clin Appl Thromb. 2020;26:1076029620955240. 10.1177/1076029620955240 PMC7476329 32873056

[8] Ballas SK , Lieff S , Benjamin LJ , Dampier CD , Heeney MM , Hoppe C , et al. Definitions of the phenotypic manifestations of sickle cell disease. Am J Hematol. 2010;85(1):6–13. 19902523 10.1002/ajh.21550 PMC5046828

[9] Vichinsky EP , Styles LA , Colangelo LH , Wright EC , Castro O , Nickerson B . Acute chest syndrome in sickle cell disease: clinical presentation and course. Cooperative Study of Sickle Cell Disease. Blood 1997;89(5):1787–92. 9057664

[10] Maitre B , Habibi A , Roudot‐Thoraval F , Bachir D , Belghiti DD , Galacteros F , et al. Acute chest syndrome in adults with sickle cell disease. Chest 2000;117(5):1386–92. 10807826 10.1378/chest.117.5.1386

[11] Nur E , Gaartman AE , van Tuijn CFJ , Tang MW , Biemond BJ . Vaso‐occlusive crisis and acute chest syndrome in sickle cell disease due to 2019 novel coronavirus disease (COVID‐19). Am J Hematol. 2020;95(6):725–6. 32267016 10.1002/ajh.25821 PMC7262303

[12] Beerkens F , John M , Puliafito B , Corbett V , Edwards C , Tremblay D . COVID‐19 pneumonia as a cause of acute chest syndrome in an adult sickle cell patient. Am J Hematol. 2020;95:E154–6. 32243621 10.1002/ajh.25809

[13] Hussain FA , Njoku FU , Saraf SL , Molokie RE , Gordeuk VR , Han J . COVID‐19 infection in patients with sickle cell disease. Br J Haematol. 2020;189:851–2. 32314798 10.1111/bjh.16734 PMC7264585

[14] McCloskey KA , Meenan J , Hall R , Tsitsikas DA . COVID‐19 infection and sickle cell disease: a UK centre experience. Br J Haematol. 2020;190:e57–8. 32369606 10.1111/bjh.16779

[15] De Luna G , Habibi A , Deux J‐F , Colard M , Pham Hung d'Alexandry d'Orengiani A‐L , Schlemmer F , et al. Rapid and severe Covid‐19 pneumonia with severe acute chest syndrome in a sickle cell patient successfully treated with tocilizumab. Am J Hematol. 2020;95(7):876–8. 32282956 10.1002/ajh.25833 PMC7262334

[16] Odièvre M‐H , de Marcellus C , Ducou Le Pointe H , Allali S , Romain A‐S , Youn J , et al. Dramatic improvement after tocilizumab of severe COVID‐19 in a child with sickle cell disease and acute chest syndrome. Am J Hematol. 2020;95:E192–4. 32358817 10.1002/ajh.25855 PMC7267654

[17] Huang C , Wang Y , Li X , Ren L , Zhao J , Hu Y , et al. Clinical features of patients infected with 2019 novel coronavirus in Wuhan, China. Lancet 2020;395(10223):497–506. 10.1016/S0140-6736(20)30183-5 31986264 PMC7159299

[18] Mahat RK , Panda S , Rathore V , Swain S , Yadav L , Sah SP . The dynamics of inflammatory markers in coronavirus disease‐2019 (COVID‐19) patients: a systematic review and meta‐analysis. Clin Epidemiol Glob Heal. 2021;11:100727. https://pubmed.ncbi.nlm.nih.gov/33778183 10.1016/j.cegh.2021.100727 PMC7979575 33778183

[19] Zeng F , Huang Y , Guo Y , Yin M , Chen X , Xiao L , et al. Association of inflammatory markers with the severity of COVID‐19: a meta‐analysis. Int J Infect Dis. 2020;96:467–74. https://www.sciencedirect.com/science/article/pii/S1201971220303623 32425643 10.1016/j.ijid.2020.05.055 PMC7233226

[20] Rees DC , Gibson JS . Biomarkers in sickle cell disease. Br J Haematol. 2012;156(4):433–45. 10.1111/j.1365-2141.2011.08961.x 22122125

[21] Damanhouri GA , Jarullah J , Marouf S , Hindawi SI , Mushtaq G , Kamal MA . Clinical biomarkers in sickle cell disease. Saudi J Biol Sci. 2015;22(1):24–31. 25561879 10.1016/j.sjbs.2014.09.005 PMC4281636

[22] Koduri PR . Iron in sickle cell disease: a review why less is better. Am J Hematol. 2003;73(1):59–63. 12701123 10.1002/ajh.10313

[23] Dignass A , Farrag K , Stein J . Limitations of serum ferritin in diagnosing iron deficiency in inflammatory conditions. Int J chronic Dis. 2018;2018:9394060. 29744352 10.1155/2018/9394060 PMC5878890

[24] Makani J , Tluway F , Makubi A , Soka D , Nkya S , Sangeda R , et al. A ten year review of the sickle cell program in Muhimbili National Hospital, Tanzania. BMC Hematol. 2018;18(1):33. 10.1186/s12878-018-0125-0 30459954 PMC6236876

[25] Mangosongo B , Kalokola F , Munubhi E , Mpembeni R . Iron deficiency in sickle cell anaemia patients in Dar es Salaam, Tanzania. Tanzania Med J. 2007;19:6–9.

[26] Kassim A , Thabet S , al‐Kabban M , Al‐Nihari K . Iron deficiency in Yemeni patients with sickle‐cell disease. East Mediterr Health J. 2012;18(3):241–5. 22574477 10.26719/2012.18.3.241

[27] Melton CW , Haynes JJ . Sickle acute lung injury: role of prevention and early aggressive intervention strategies on outcome. Clin Chest Med. 2006;27(3):487–502, vii. 16880058 10.1016/j.ccm.2006.04.001

[28] Swerdlow PS . Red cell exchange in sickle cell disease. Hematology 2006;2006(1):48–53. 10.1182/asheducation-2006.1.48 17124039

[29] Chamba C , Iddy H , Tebuka E , Tluway F , Osati E , Budodi N , et al. Limited exchange transfusion can be very beneficial in sickle cell anemia with acute chest syndrome: a case report from Tanzania. Case Rep Hematol. 2018;2018:5253625. 10.1155/2018/5253625 30034890 PMC6032970

[30] Darbari DS , Fasano RS , Minniti CP , Castro OO , Gordeuk VR , Taylor JG 6th , et al. Severe vaso‐occlusive episodes associated with use of systemic corticosteroids in patients with sickle cell disease. J Natl Med Assoc. 2008;100(8):948–51. 28643632 10.1016/S0027-9684(15)31410-3 PMC5577928

[31] Hematology AS . COVID‐19 and sickle cell disease: frequently asked questions. 2021. Accessed October 13, 2021. Available from: https://www.hematology.org/covid‐19/covid‐19‐and‐sickle‐cell‐disease

[32] Steinberg MH , McCarthy WF , Castro O , Ballas SK , Armstrong FD , Smith W , et al. The risks and benefits of long‐term use of hydroxyurea in sickle cell anemia: a 17.5 year follow‐up. Am J Hematol. 2010;85(6):403–8. 20513116 10.1002/ajh.21699 PMC2879711

